# Muscle Fn14 gene expression is associated with fat‐free mass retention during energy deficit at high altitude

**DOI:** 10.14814/phy2.13801

**Published:** 2018-07-16

**Authors:** Stefan M. Pasiakos, Claire E. Berryman, John W. Carbone, Nancy E. Murphy, Christopher T. Carrigan, Marcas M. Bamman, Arny A. Ferrando, Andrew J. Young, Lee M. Margolis

**Affiliations:** ^1^ Military Nutrition Division U.S. Army Research Institute of Environmental Medicine Natick Massachusetts; ^2^ Oak Ridge Institute of Science and Education Oak Ridge Tennessee; ^3^ School of Health Sciences Eastern Michigan University Ypsilanti Michigan; ^4^ Department of Cell, Developmental, and Integrative Biology University of Alabama at Birmingham Birmingham Alabama; ^5^ Department of Geriatrics The Center for Translational Research in Aging & Longevity Donald W. Reynolds Institute of Aging University of Arkansas for Medical Sciences Little Rock Arkansas

**Keywords:** Hypoxia, interleukin‐6, myogenin, negative energy balance, TWEAK

## Abstract

Intramuscular factors that modulate fat‐free mass (FFM) loss in lowlanders exposed to energy deficit during high‐altitude (HA) sojourns remain unclear. Muscle inflammation may contribute to FFM loss at HA by inducing atrophy and inhibiting myogenesis via the tumor necrosis factor (TNF)‐like weak inducer of apoptosis (TWEAK) and its receptor, fibroblast growth factor‐inducible protein 14 (Fn14). To explore whether muscle inflammation modulates FFM loss reportedly developing during HA sojourns, muscle inflammation, myogenesis, and proteolysis were assessed in 16 men at sea level (SL) and following 21 days of energy deficit (−1862 ± 525 kcal/days) at high altitude (HA, 4300 m). Total body mass (TBM), FFM, and fat mass (FM) were assessed using DEXA. Gene expression and proteolytic enzymatic activities were assessed in muscle samples collected at rest at SL and HA. Participants lost 7.2 ± 1.8 kg TBM (*P *<* *0.05); 43 ± 30% and 57 ± 30% of the TBM lost was FFM and FM, respectively. Fn14, TWEAK, TNF alpha‐receptor (TNF *α*‐R), TNF *α*, MYOGENIN, and paired box protein‐7 (PAX7) were upregulated (*P* < 0.05) at HA compared to SL. Stepwise linear regression identified that Fn14 explained the highest percentage of variance in FFM loss (*r* ^*2*^ **= 0.511, *P *<* *0.05). Dichotomization of volunteers into HIGH and LOW Fn14 gene expression indicated HIGH lost less FFM and more FM (28 ± 28% and 72 ± 28%, respectively) as a proportion of TBM loss than LOW (58 ± 26% and 42 ± 26%; *P* < 0.05) at HA. MYOGENIN gene expression was also greater for HIGH versus LOW (*P* < 0.05). These data suggest that heightened Fn14 gene expression is not catabolic and may protect FFM during HA sojourns.

## Introduction

Hypoxia exposure during high‐altitude (HA) sojourns elicits a persistent systemic inflammatory response (Mazzeo et al. 1985; Hartmann et al. 2000; Lundby and Steensberg 2004). This response represents a homeostatic adaptation to hypoxia that may promote acute‐phase protein synthesis, angiogenesis, and erythropoiesis (Mazzeo et al. 1985; Klausen et al. 1997; Mazzeo 2005). Sojourners at HA are also subject to additional pro‐inflammatory stressors, such as high physical activity levels (Hoppeler et al. 1990; Hoyt et al. 1994) and energy deficits (Butterfield et al. 1985; Rose et al. 1985), that may exacerbate inflammation and/or modulate its downstream effects. Strenuous military operations at sea level (SL) induce an inflammatory response that upregulates the hepatic release of hepcidin, a regulator of iron status, associated, at least in part, with failure to match total daily energy expenditure with sufficient energy intake (McClung et al. 2013; Pasiakos et al. 2016). The muscle inflammatory response associated with energy deficits may be due to a reduction in muscle energy availability (Hennigar et al. 2017). The magnitude and consequences of the inflammation caused during military operations have received only limited attention (McClung et al. 2013; Pasiakos et al. 2016), particularly for operations at HA (Hagobian et al. 2006). The skeletal muscle inflammatory response might be a mechanism modulating loss of muscle mass often experienced by lowlanders sojourning at HA (Pasiakos et al. 2017).

Muscle inflammation can promote muscle loss (Dogra et al. 2006; Mittal et al. 2010; Merritt et al. 2013). This generally occurs in response to injury and pathogenic conditions of muscle wasting, as chronic elevations and/or exceedingly high muscle inflammation can inhibit muscle regeneration (Merritt et al. 1985; Dogra et al. 2007a), blunt muscle protein synthesis (Bamman et al. 2015), and contribute to muscle atrophy (Kumar et al. 2012; Merritt et al. 2012, 2013). This catabolic state is modulated, in part, by TNF alpha (TNF*α*), the TNF‐like weak inducer of apoptosis (TWEAK) and its receptor, fibroblast growth factor‐inducible 14 (Fn14), which activate nuclear factor‐*κ*B (NF‐*κ*B), subsequent atrophic gene expression, and inhibition of muscle regeneration (Dogra et al. 2006, 2007a). We suspect that because FFM is often markedly degraded when lowlanders are chronically underfed when sojourning at HA that muscle inflammation will be upregulated, triggering downstream atrophic gene expression, and inhibition of muscle regeneration. However, to the best of our knowledge, no study has investigated the TWEAK‐Fn14 axis response or examined associated downstream effects on muscle mass (i.e., fat‐free mass; FFM) in lowlanders exposed to the combined stress of HA and energy deficit.

The objective of this study was to determine the combined effects of HA exposure and energy deficit on muscle inflammation and examine associations between markers of inflammation and muscle mass lost (Berryman et al. 2018). This secondary analysis was pursued to explore potential intramuscular explanations for the interindividual variability and magnitude of FFM lost we observed in lowlanders exposed to 21 days of energy deficit at HA as part of the parent study (Berryman et al. 2018). We hypothesized that muscle inflammation would increase in response to the combined stress of hypoxia and energy deficit, and the magnitude of this response would be directly associated with the magnitude of FFM loss experienced by individuals exposed to the stress of hypoxic exposure and energy deficit.

## Methods

### Participants

Participant eligibility, recruitment details, and results of the primary study objective have been previously reported elsewhere (Berryman et al. 2018). Due to limited quantity of muscle sample for one volunteer, data from 16 of the original 17 male lowlanders that completed the parent study are included in this report (Berryman et al. 2018). All volunteers provided written informed consent prior to participation. This study was approved by the Institutional Review Board at the US Army Research Institute of Environmental Medicine (USARIEM, Natick, MA) and registered at http://www.clinical trials.gov as NCT02731066. Investigators adhered to the policies for protection of human subjects as described in the US Department of Defense Instruction 3216.02, and the research was conducted in adherence with the provisions of 32 CFR Part 219.

### Experimental design

**Figure 1:**
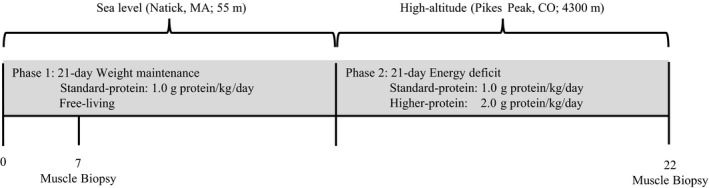
Study design. Energy deficit was designed to be 40% of total energy needs; however, on the basis of changes in body energy stores, the actual deficit was closer to 70% (−1862 ± 525 kcal/day). The current analysis was part of a larger study that assessed the impact of dietary protein intake on changes in body composition in response to 21 days of energy deficit at high altitude (4300 m). Habitual protein intake did not influence end point measures in this analysis, so diet groups were collapsed.

This diet and physical activity controlled study consisted of 21 days at SL (Natick, MA; 55 m; Fig. 1) followed immediately by 22 days at HA (Pikes Peak, CO; 4300 m) (Berryman et al. 2018). Participants maintained their habitual exercise routines and adhered to strict dietary instruction at SL, and were weight stable (Berryman et al. 2018). At HA, additional exercise was prescribed daily and the participants were fed an energy deficient diet. Skeletal muscle inflammation and downstream myogenic regulatory factors were assessed in vastus lateralis biopsy samples collected under fasted, rested conditions on day 7 at SL and on day 22 at HA. The SL muscle biopsies were performed on day 7 in order to provide sufficient time to pack and transport laboratory equipment from our laboratory in Natick, MA, to our laboratory on Pikes Peak, CO.

After completing the SL phase, participants were flown from Boston, MA to Denver, CO and driven to Colorado Springs, CO, where they stayed overnight under supervision in a local hotel. The following morning (HA day 1, ~3 am), participants were transported by car to the USARIEM Maher Memorial Laboratory on the summit of Pikes Peak, CO and underwent additional experimental procedures reported elsewhere (Berryman et al. 2018). Starting on HA day 2, participants were randomized to a controlled diet with either standard‐ (mean ± SD, 1.1 ± 0.0 g protein·kg^−1^ day^−1^, 2.8 ± 0.4 g carbohydrate·kg^−1^ day^−1^, 1.1 ± 0.1 g fat·kg^−1^ day^−1^, and 1950 ± 186 kcal day^−1^) or higher‐ (2.1 ± 0.0 g protein kg^−1^ day^−1^, 3.0 ± 0.2 g kg^−1^ day^−1^, 0.7 ± 0.1 g fat kg^−1^ day^−1^, and 1894 ± 286 kcal day^−1^) protein for the next 21 days (Berryman et al. 2018).

To induce weight loss, exercise‐induced energy expenditure (EIEE) was increased by 10% above estimated SL total daily energy expenditures (TDEE) [i.e., HA EIEE = SL EIEE + (0.1 ×  SL TDEE)], and daily energy intake was decreased by 30% relative to TDEE estimated at SL to maintain body mass to induce an energy deficit level consistent with our previous work (McClung et al. 2013; Pasiakos et al. 2013). As previously described (Berryman et al. 2018), SL TDEE was estimated from the sum of resting metabolic rate measured by indirect calorimetry (True Max 2400, Parvo Medics, Sandy, UT, USA; multiplied by 1.3 to account for activities of daily living and diet induced thermogenesis) and EIEE estimated from corresponding metabolic equivalents of activities reported in 3 days physical activity logs. These data were then averaged with TDEE derived from the Harris‐Benedict equation (Roza and Shizgal 1984), and activity derived from 3‐days records. Exercise was performed daily at HA, with the exception of day 7, 14, and 21, the day prior to the muscle biopsy procedure on day 22 at HA (exercise was also restricted for the 24 h preceding the SL biopsy). Exercise was supervised and consisted of treadmill walking, running, and outdoor hiking, with the energy cost estimated using the Pandolf et al. (1977) Equation, with adjustments for body mass loss, terrain factors, and downhill walking (Pimental and Pandolf 1979).

Body mass was measured daily at HA and body composition (total body mass, TBM; FFM; and fat mass, FM) was determined at baseline, before ascent to HA (SL day 20), and immediately after descent from HA (~0630–0800 on HA day 23) using dual‐energy X‐ray absorptiometry (DEXA; iDEXA, GE Lunar Corporation, Madison, WI, USA) (Berryman et al. 2018). In this study, FFM refers to TBM minus both FM and bone mass. As previously reported (Berryman et al. 2018), TBM loss was more than expected for an estimated, prescribed energy deficit of 40%. As such, changes in body energy stores were used to better estimate the total daily energy deficit (Hoyt et al. 2006; Berryman et al. 2018). Those estimates are presented in this paper for descriptive purposes and to explore the role of energy balance on modulating muscle inflammation.

### Muscle inflammatory and myogenic mRNA

Muscle interleukin 6 (IL‐6), IL‐6 receptor (IL‐6R), TNF*α*, TNF*α*‐receptor (TNF*α*‐R), TWEAK, Fn14, paired box protein‐7 (PAX7), MYOD, MYOGENIN, MuRF1, and ATROGIN‐1 gene expression were determined using primers from Bio‐Rad Laboratories (Hercules, CA, USA). TRIzol (ThermoFisher, Waltham, MA, USA) was used to isolate total RNA from 20 mg of muscle followed by quantity and quality assessments using a Nanodrop ND‐2000 spectrophotometer (ThermoFisher). cDNA was then synthesized from 500 ng of total RNA using iScriptTM Advanced cDNA Synthesis Kits from Bio‐Rad Laboratories and a T100™ Thermal Cycler (Bio‐Rad). cDNA amplifications were performed using the StepOnePlus Real‐Time PCR System (Applied Biosystems, Foster City, CA, USA). Samples were run in 20 *μ*L reactions in duplicate using iTaq™ Universal SYBR^®^ Green Supermix (Bio‐Rad). mRNA data were normalized to the geometric mean of *β*‐actin, GAPDH, and B2M mRNA, and fold changes (HA vs. SL) were calculated using the ΔΔC_*T*_ method (Pfaffl 2001). The MURF1 and ATROGIN‐1 gene expression responses to the HA and severe energy deficit have been reported previously (Margolis et al. 2018), but are reported briefly in this paper to explore the potential role of muscle inflammation in mediating MURF1 and ATROGIN‐1 gene expression.

### NF‐*κ*B phosphorylation and total protein expression

Phosphorylation status and total protein content of NF‐*κ*B p65 were determined using standard homogenization and Western blot methodologies, as described in detail elsewhere (Margolis et al. 2016). In brief, after 15 *μ*g of total protein was separated by SDS‐PAGE and transferred to polyvinylidene fluoride membranes, primary antibodies for p‐NF‐*κ*B p65^Ser468^ and total NF‐*κ*B p65 (Cell Signaling Technology, Danvers, MA, USA) were applied, and membranes were incubated overnight at 4°C. Secondary antibody (anti‐rabbit IgG conjugate with horseradish peroxidase; Cell Signaling Technology) and chemiluminescent reagent (Pierce Biotechnology, Rockford, IL, USA) were applied the next morning, and blots were quantified using the ChemiDoc XRS and Image Lab software from Bio‐Rad Laboratories. Heat shock protein 90 (HSP90) was used to confirm equal protein loading across wells. Phosphorylation and total protein content of NF‐*κ*B p65 were normalized to HSP90, the ratio of phosphorylation‐to‐total protein was determined, and HA data were expressed as fold change relative to SL.

### 26S proteasome enzymatic activity

26S proteasome responses to HA and severe energy deficit have been reported previously (Margolis et al. 2018), but included in this paper to explore the potential role of muscle inflammation in mediating this response. Extended methodological details for assessing 26S *β*_5_ (chymotrypsin‐like), 26S *β*_2_ (trypsin‐like), and 26S *β*_1_ (peptidyl‐glutamyl peptide hydrolyzing [PGPH]‐like) enzymatic activities have been reported (Carbone et al. 2013). HA proteolytic activity data were expressed as fold change relative to SL.

### Statistical analysis

This study was a secondary objective of a larger randomized controlled trial powered to detect differences in FFM between standard‐ and higher‐protein diets following HA exposure and concomitant energy deficit (Berryman et al. 2018). As reported, dietary protein intake had no effect on FFM in response to the combined physiological and environmental stress (Berryman et al. 2018). Therefore, we did not expect dietary protein group to affect skeletal muscle inflammation, regeneration, and proteasome enzymatic activities after 21 days of exposure to HA and energy deficit. After testing for normality (Shapiro‐Wilk), and log_2_ transformation of skewed data (all mRNA endpoints), we examined the effects of dietary protein on study outcomes at HA relative to SL using repeated measures ANOVA with phase (SL and HA) and dietary protein group (standard‐ and higher‐protein) as within‐ and between‐subjects factors, respectively. As anticipated, there were no effects of dietary protein group on any study outcome.

The data were then re‐analyzed, excluding dietary protein group as a between‐subjects factor, using paired *t*‐tests (HA vs. SL). To be consistent with Bamman et al. (2015), stepwise linear regression was used to explore the associations between skeletal muscle inflammatory markers and FFM loss, expressed as the proportion of TBM loss in response to HA and severe energy deficit. Participants were then dichotomized as HIGH and LOW based on median gene expression at HA relative to SL for the inflammatory marker (i.e., Fn14) most strongly associated with FFM loss. Body composition and myogenic gene expression responses to HA exposure and severe energy deficit were then analyzed by HIGH and LOW group using unpaired *t*‐tests. Within the text, data are presented as mean ± SD; in the figures, data are presented as mean ± SD and as individual data points to demonstrate intersubject variability. The *α* level for significance was two‐tailed and set at *P* < 0.05, and data were analyzed using IBM SPSS Statistics for Windows Version 22.0 (IBM Corp. Armonk, NY, USA).

## Results

Participants (*n* = 16) lost 7.2 ± 1.8 kg TBM (SL, 79.8 ± 14.4 kg; HA, 72.6 ± 13.1 kg) and 43 ± 30% (3.5 ± 2.5 kg) and 57 ± 30% (3.7 ± 1.4 kg) of the TBM lost was FFM and FM, respectively. Daily exercise contributed 671 ± 172 kcal/day (108 ± 23 min/day) and the estimated daily energy deficit based on changes in body energy stores was 1862 ± 525 kcal/day.

**Figure 2:**
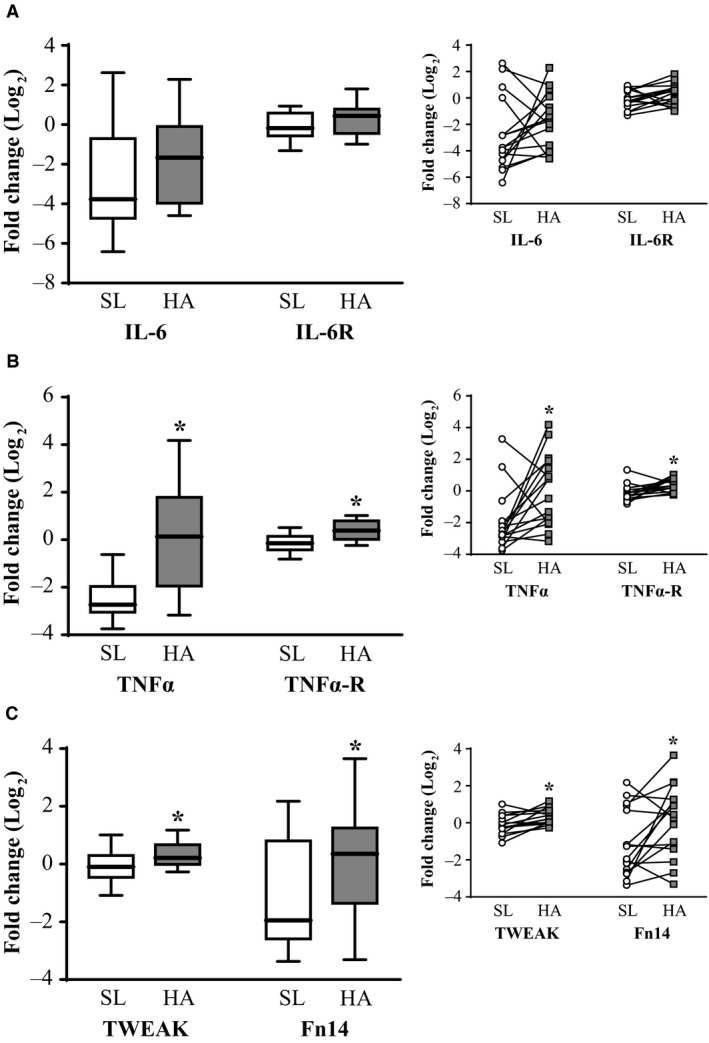
Skeletal muscle IL‐6 and IL‐6R (A), TNF*α* and TNF*α*‐R (B), and TWEAK and Fn14 (C) gene expression in male (*n* = 16) lowlanders exposed to 21 days of energy deficit at high altitude (4300 m). Data were normalized to the geometric mean of *β*‐actin, GAPDH, and B2M gene expression, and fold changes were calculated using the ΔΔC_*T*_ method (Pfaffl 2001). HA data were expressed as fold change relative to SL to examine differences using paired *t*‐tests after data were log_2_ transformed. Values are presented as mean ± SD and individually per participant. *HA different than SL, *P* < 0.05. IL‐6, interleukin 6; IL‐6R, interleukin 6 receptor; TNF*α*, tumor necrosis factor alpha; TNF*α*‐R, tumor necrosis factor alpha‐receptor; TWEAK, tumor necrosis factor‐like weak inducer of apoptosis; Fn14, fibroblast growth factor‐inducible 14; SL, day 7 at sea level; and HA, day 22 at high altitude.

**Figure 3:**
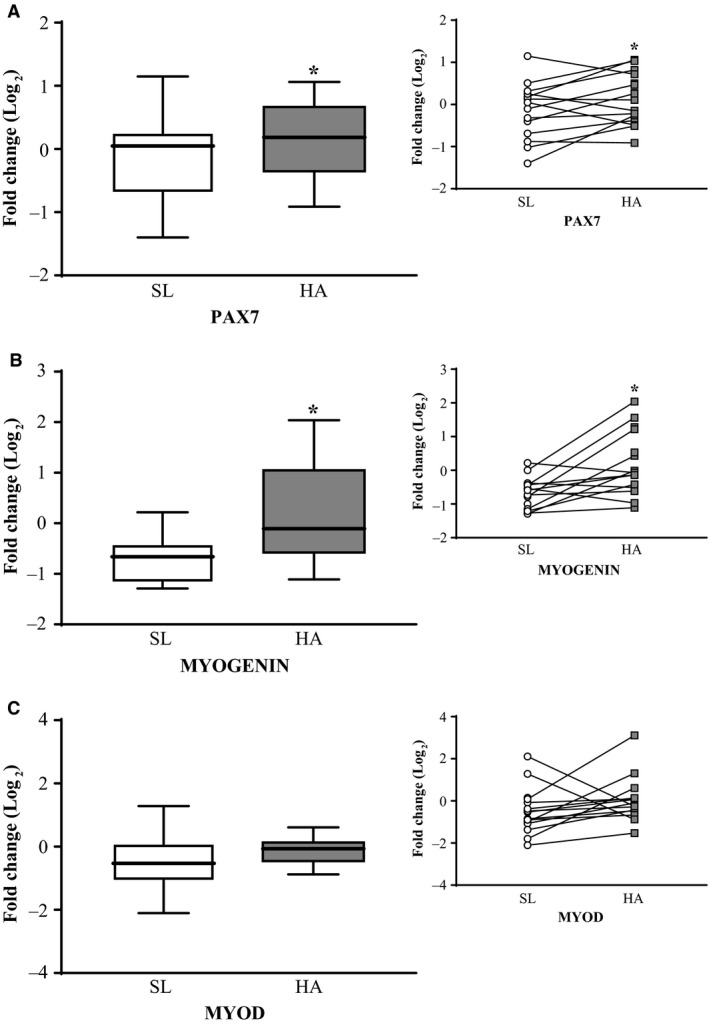
Skeletal muscle PAX7 (A), MYOGENIN (B), and MYOD (C) gene expression in male (*n* = 16) lowlanders exposed to 21 days of energy deficit at high altitude (4300 m). Data were normalized to the geometric mean of *β*‐actin, GAPDH, and B2M gene expression, and fold changes were calculated using the ΔΔC_*T*_ method (Pfaffl 2001). HA data were expressed as fold change relative to SL to examine differences using paired *t*‐tests after data were log_2_ transformed. Values are presented as mean ± SD and individually per participant. *HA different than SL, *P* < 0.05. PAX7, paired box protein‐7; SL, day 7 at sea level; and HA, day 22 at high altitude.

Muscle IL‐6 and IL‐6R gene expressions were not significantly different at HA relative to SL (Fig. 2A). TNF*α*, TNF*α*‐R, TWEAK, and Fn14 gene expressions were upregulated at HA relative to SL (Fig. 2B–C, *P *< 0.05), while p‐NF‐*κ*B p65^Ser468^ or NF‐*κ*B p65 total protein content at HA was not significantly different from SL. PAX7 and MYOGENIN gene expression were upregulated at HA relative to SL (Fig. 3A–B, *P* < 0.05), but MYOD gene expression was not significantly different between HA and SL (Fig. 3C). MuRF1 and ATROGIN‐1 gene expression were not significantly different at HA relative to SL. Likewise, there were no significant differences in chymotrypsin‐like, trypsin‐like, and PGPH‐like enzymatic activities at HA compared to SL.

**Figure 4:**
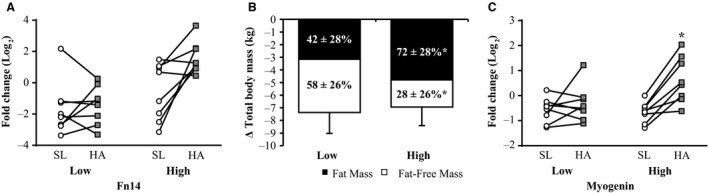
Fn14 (A), FM and FM loss as a proportion (%) of total body mass loss (B) and MYOGENIN gene expression (C) in male (*n* = 16) lowlanders dichotomized based on median Fn14 gene expression at HA relative to SL into LOW and HIGH after being exposed to 21 days of energy deficit at high altitude (4300 m). FFM and FM differences between HIGH and LOW were determined using unpaired *t*‐tests. MYOGENIN gene expression data were normalized to the geometric mean of *β*‐actin, GAPDH, and B2M gene expression, and fold changes were calculated using the ΔΔC_*T*_ method (Pfaffl 2001). HA data were expressed as fold change relative to SL to examine differences between HIGH and LOW using unpaired *t*‐tests after mRNA data were log_2_ transformed. Values are presented as mean ± SD and individually per participant. *HIGH significantly different from LOW, *P* < 0.05. FFM, fat‐free mass; FM, fat mass; SL, day 7 at sea level; and HA, day 22 at high altitude.

Stepwise linear regression analyses showed increased IL‐6 (*r *=* *−0.605), TNF*α* (*r *=* *−0. 496), TWEAK (*r *=* *−0.513), and Fn14 (*r *=* *−0.715) gene expression were associated (*P* < 0.05) with less FFM loss. Based on these results, participants were dichotomized into HIGH and LOW according to the median Fn14 gene expression at HA, respectively (Fig. 4A). HIGH lost less FFM and more FM (28 ± 28% and 72 ± 28%), as a proportion of TBM loss, than LOW (58 ± 26% and 42 ± 26%; Fig. 4B, *P* < 0.05). MYOGENIN gene expression after HA exposure and severe energy deficit was also greater for HIGH versus LOW (Fig. 4C, *P* < 0.05). MYOGENIN gene expression changes in response to HA and severe energy deficit were associated *(r *=* *0.530, *P* < 0.05) with the change in Fn14. There were no significant differences in MuRF1 or ATROGIN‐1 gene expression, or chymotrypsin‐like, trypsin‐like, and PGPH‐like enzymatic activities between HIGH and LOW. No significant differences were observed in EIEE between the two groups (HIGH: 686 ± 156 kcal day^−1^, LOW: 655 ± 196 kcal day^−1^, *P *>* *0.05) or in the estimated energy deficit (HIGH: ‐2021 ± 228 kcal day^−1^, LOW: −1702 ± 693 kcal day^−1^, *P *>* *0.05).

## Discussion

The current study explored muscle inflammation, myogenic regulatory factor gene expression, and ubiquitin‐mediated proteolytic responses to the 9% loss of TBM induced by the combined stress of chronic HA exposure and concomitant energy deficit. The primary findings show that, in response to 21 days of severe energy deficit at HA: (1) muscle inflammation and myogenic regulatory factor gene expression were upregulated, with no measureable change in ubiquitin‐mediated proteolytic activity; and (2) an increase in the inflammatory response, particularly Fn14, was associated with the upregulation of MYOGENIN and attenuation of FFM loss. These findings suggest that, contrary to our hypothesis, heightened Fn14 gene expression was not catabolic and may protect FFM during energy deficit at HA by upregulating myogenesis.

Certain experimental issues must be acknowledged to appropriately interpret our findings and their potential implications. First, our study design did not include a SL control group that experienced the energy deficit and a HA control group that experienced energy balance. This precludes us from delineating the independent effects of the sustained underfeeding and stress of HA on muscle inflammatory gene expression and its associated effects. Also, while our analytical approach was modeled after recent studies exploring the role of muscle inflammation on indices of muscle anabolism, wasting, and function (Merritt et al. 1985, 2013; Bamman et al. 2015; Yarar‐Fisher et al. 2016), the observational and correlative nature of our analysis may be considered limitations. However, we show muscle inflammation, particularly Fn14, is upregulated and associated with greater MYOGENIN and reduced FFM loss following 21 days of energy deficit at HA, which are all novel findings that may advance our current understanding of how healthy muscle adapts to energy deficit and the environmental stress of unaccustomed HA exposure.

Muscle inflammation and its potential downstream effects on proteolytic activity in healthy individuals exposed to chronic HA and concomitant underfeeding, to our knowledge, has not been explored. We observed modest increases in muscle TNF*α*, TNF*α*‐R, TWEAK, and Fn14 gene expression in individuals that lost 3.5 ± 2.5 kg of FFM (43 ± 30% of TBM lost). Upregulated TNF‐*α* and TWEAK gene expression may indirectly induce skeletal muscle atrophy by activating NF‐*κ*B signaling and subsequent transcription of the ubiquitin ligases, ATROGIN‐1 and MuRF1 (Cai et al. 2004; Dogra et al. 2006, 2007a). However, despite increases in TNF‐*α* and TWEAK gene expression, NF‐*κ*B signaling, and downstream ATROGIN‐1 and MuRF1 gene expression were not different after 21 days of energy deficit at HA. More importantly, there were no changes in proteolytic activity, as 26S proteasome chymotrypsin‐like, trypsin‐like, and PGPH‐like enzymatic activities after chronic HA exposure and energy deficit were not different than SL. Overall, these findings suggest that regardless of the observed muscle inflammatory response, healthy muscle is relatively protected against excessive muscle protein catabolism in response to chronic HA exposure and concomitant energy deficit.

Although NF‐*κ*B signaling and subsequent proteolysis were not affected by the combined stress of HA and energy deficit, myogenesis was, as PAX7 and MYOGENIN were upregulated following 21 days of energy deficit at HA. Although inflammation is generally associated with muscle degradation, transient elevation in muscle inflammation following routine exercise is a normal physiological response to contractile‐mediated muscle damage that triggers muscle remodeling. Specifically, elevations in muscle inflammation in response to acute exercise stress occur with parallel increases in myogenic regulatory factor gene expression to aid in the regeneration of damaged muscle fibers (Raue et al. 1985a,b; Chen et al. 2007). TWEAK and its receptor, Fn14, may be the link between muscle inflammation and regeneration (Girgenrath et al. 2006), whereby TWEAK stimulates myoblast proliferation and Fn14 is required for myoblast differentiation into myofibers (Girgenrath et al. 2006; Dogra et al. 2007b). Adult muscle regeneration that is mediated by myogenic regulatory factors, a form of nonhypertrophic remodeling, results in the repair or replacement of damaged muscle fibers (Tedesco et al. 2010; Burd and De Lisio 2017). This process of regeneration is critical for the maintenance of muscle mass. Raue et al. (1985a) showed that a 30 min treadmill run at 75% of max V̇O_2_ (i.e., ~3.4 L/min, ~506 kcal/30 min) elicits an 8.5 to 12‐fold increase in Fn14 gene expression 12–24 h postexercise. In the current study, exercise was prohibited for at least 36 h prior to the muscle biopsy, but on the other days at HA participants were required to complete multiple bouts (Bamman et al. 2015; Burd and De Lisio 2017; Berryman et al. 2018) of aerobic‐type exercise to elicit an EIEE of approximately 670 kcal/day. The observed increase in Fn14 and associated myogenic regulatory factors following 21 day of energy deficit at HA was likely a residual and/or cumulative response to repeated exercise bouts performed at least 36 h before muscle samples were collected. Our findings suggest that magnitude of muscle inflammation observed, whether due to chronic exposure to HA and severe underfeeding or multiple bouts of daily aerobic exercise, may benefit muscle maintenance by favoring regeneration.

Based on a previous study by Bamman et al. (2015), we used regression analysis and subsequent dichotomization to explore whether the interindividual variability in muscle inflammation dictated the magnitude of FFM loss in response to energy deficit at HA. In the Bamman study (Bamman et al. 2015), patients undergoing elective total hip arthroplasty were dichotomized based on perioperative Fn14 gene expression in the gluteal muscle of the surgical limb, and deemed either susceptible (high Fn14 expression relative to controls) or not susceptible (low Fn14 gene expression relative to controls) to excessive muscle inflammation. Patients with high Fn14 gene expression had lower muscle protein synthesis rates and higher NF‐*κ*B activity and MURF1 gene expression compared to patients with lower Fn14 gene expression (Bamman et al. 2015). Bamman et al. (2015) concluded that elevated Fn14 gene expression may be a prognostic indicator of poor recovery from surgery. Accordingly, we expected that the muscle inflammatory response to 21 days of energy deficit at HA would be detrimental, such that those with an elevated muscle inflammatory response would lose more FFM than participants exhibiting minimal changes in muscle inflammation. After regression analysis confirmed Fn14 was the strongest predictor of FFM loss, participant dichotomization into HIGH and LOW Fn14 demonstrated that those classified as HIGH lost significantly less FFM and more FM than those classified as LOW. We suspect that the apparent beneficial effect of increased muscle inflammation, particularly Fn14, in the current study was due to the upregulation in myogenic regulatory factor gene expression. This is highlighted by the finding that MYOGENIN gene expression in response to chronic HA and concomitant energy deficit was greater in HIGH than LOW. These results, although in contrast to our original hypothesis, are in accordance with studies that show muscle inflammation in response to routine exercise is not overtly catabolic and aids in the repair, remodeling, and maintenance of healthy muscle tissue (Raue et al. 1985a,b; Warren et al. 2002; Karalaki et al. 2009).

In conclusion, 21 days of HA exposure with concomitant energy deficit, achieved with high levels of aerobic‐type activity and dietary restriction, upregulated muscle inflammation. The modest upregulation in muscle inflammation occurred without any change in muscle proteolysis. However, myogenic regulatory factor gene expression was upregulated. Participants that exhibited a greater increase in Fn14 gene expression during chronic HA and energy deficit exposure had greater expression of the myogenic regulatory factor MYOGENIN and lost less FFM than participants who had a less robust inflammatory response. These data suggest a heightened muscle inflammatory response to physiological (i.e., exercise and diet restriction) and environmental stress promotes a myogenic response that may protect muscle mass by aiding in muscle regeneration.

## Conflict of Interest

The opinions or assertions contained herein are the private views of the authors and are not to be construed as official or as reflecting the views of the Army or the Department of Defense. Any citations of commercial organizations and trade names in this report do not constitute an official Department of the Army endorsement of approval of the products or services of these organizations. The authors declare that they have no conflicts of interest.
